# mRNA and lncRNA co-expression network in mice of acute intracerebral hemorrhage

**DOI:** 10.3389/fnmol.2023.1166875

**Published:** 2023-04-28

**Authors:** Zhe Yu, En Hu, Yiqing Cai, Wenxin Zhu, Quan Chen, Teng Li, Zhilin Li, Yang Wang, Tao Tang

**Affiliations:** ^1^Department of Integrated Traditional Chinese and Western Medicine, Institute of Integrative Medicine, Xiangya Hospital, Central South University, Changsha, Hunan, China; ^2^National Clinical Research Center for Geriatric Disorders, Xiangya Hospital, Central South University, Changsha, Hunan, China

**Keywords:** mRNA, lncRNA, co-expression network, competitive endogenous RNA, intracerebral hemorrhage

## Abstract

**Background:**

Intracerebral hemorrhage (ICH) is a severe subtype of stroke lacking effective pharmacological targets. Long noncoding RNA (lncRNA) has been confirmed to participate in the pathophysiological progress of various neurological disorders. However, how lncRNA affects ICH outcomes in the acute phase is not completely clear. In this study, we aimed to reveal the relationship of lncRNA-miRNA-mRNA following ICH.

**Method:**

We conducted the autologous blood injection ICH model and extracted total RNAs on day 7. Microarray scanning was used to obtain mRNA and lncRNA profiles, which were validated by RT-qPCR. GO/KEGG analysis of differentially expressed mRNAs was performed using the Metascape platform. We calculated the Pearson correlation coefficients (PCCs) of lncRNA-mRNA for co-expression network construction. A competitive endogenous (Ce-RNA) network was established based on DIANALncBase and miRDB database. Finally, the Ce-RNA network was visualized and analyzed by Cytoscape.

**Results:**

In total, 570 differentially expressed mRNAs and 313 differentially expressed lncRNAs were identified (FC ≥ 2 and value of *p* <0.05). The function of differentially expressed mRNAs was mainly enriched in immune response, inflammation, apoptosis, ferroptosis, and other typical pathways. The lncRNA-mRNA co-expression network contained 57 nodes (21 lncRNAs and 36 mRNAs) and 38 lncRNA-mRNA pairs. The ce-RNA network was generated with 303 nodes (29 lncRNAs, 163 mRNAs, and 111 miRNAs) and 906 edges. Three hub clusters were selected to indicate the most significant lncRNA-miRNA-mRNA interactions.

**Conclusion:**

Our study suggests that the top differentially expressed RNA molecules may be the biomarker of acute ICH. Furthermore, the hub lncRNA-mRNA pairs and lncRNA-miRNA-mRNA correlations may provide new clues for ICH treatment.

## 1. Introduction

Stroke remains one of the leading causes of death worldwide and is associated with an extremely high rate of disability (Organization, G.W.H., 2022). Intracerebral hemorrhage (ICH) accounts for approximately 27.9% of all strokes, with a higher mortality than that of ischemic stroke (GBD 2019 Stroke Collaborators, 2021). Especially the mortality is as high as 30–40% in acute and subacute phases (Greenberg et al., 2022). Although surgical therapies for evacuating hematoma and alleviating the compressive effect reduce the mortality in ICH acute phase, there is no significant benefit for the long-term prognosis (Zhao et al., 2020). Apart from the drugs targeting the risk factors, there is still a lack of effective drugs for ICH patients. Therefore, the novel therapeutic targets for acute ICH need further exploration.

Long noncoding RNA (lncRNA) is a type of non-protein-coding RNA with transcripts of more than 200 nucleotides (Mattick et al., 2023). According to the lncRNA location with protein-coding genes (PCGs), lncRNA is classified into intergenic lncRNA, antisense lncRNA, and intronic lncRNA (Jarroux et al., 2017). LncRNA evolves rapidly and rarely has an ortholog in another species (Mattick et al., 2023). Although their poor conservation has confounded efforts to predict lncRNA functions across species, it has also highlighted potential functional areas (Ransohoff et al., 2018). Over the last decade, increasing attention has been paid to its functions. LncRNA contributes to translational regulation, epigenetic modifications, post-translational modifications, and protein/mRNA stability by interacting with DNA, RNA, and protein (Bridges et al., 2021). Recent studies have even found that some lncRNA encodes small peptides less than 100 aa (Choi et al., 2019). The lncRNA functions are associated with their subcellular localization and transcriptional loci relative to the modulated gene (Kopp and Mendell, 2018). Based on the biological functions previously described, lncRNA participates in multiple neurological disorders development. Accumulating evidence has implicated lncRNA dysregulation in neurodegenerative disorders, including Alzheimer’s disease (Canseco-Rodriguez et al., 2022), Parkinson’s disease (Na et al., 2022), and Huntington’s disease (Tan et al., 2021).

In ischemic stroke, some lncRNAs play the role of anti-inflammation and/or anti-apoptosis, such as MALAT1 (Zhang et al., 2017) and Nespas (Deng et al., 2019). Whereas others produce an effect of pro-inflammation and pro-inflammatory factors releasing, such as NEAT1 (Ni et al., 2020) and Gm4419 (Wen et al., 2017). In addition, lncRNA H19 (Wang et al., 2017), MEG3 (Li et al., 2020), and RMST (Sun et al., 2019) have been proven to regulate M1 microglia polarization. Although there are currently some studies on the mechanism of lncRNA in stroke, most of them merely focus on ischemic stroke. So, the mechanisms of biological functions induced by lncRNA in ICH remain scarce.

lncRNA regulation of gene transcription is also influenced by other non-coding RNA, such as microRNA (miRNA). LncRNA may competitively bind to miRNA to conduct its functions, termed competitive endogenous RNA (ce-RNA). As the sponges of miRNA, lncRNA negatively regulates the target transcripts with similar miRNA response elements (MREs). In ischemic stroke, SNHG12/LNC04080 promotes angiogenesis and blocks inflammation by interacting with miR-199a (Long et al., 2018). MALAT1 targets miR-375 and miR-145 to aggravate neurological injury through increasing reactive oxygen species (ROS) and inhibiting Aquaporin 4 (AQP4), respectively (Zhang et al., 2017; Wang et al., 2020). Currently, there is little focus on the ce-RNA in acute ICH. It is suggested that lncRNA Mtss1 promotes secondary inflammation along with miR-709 (Chen et al., 2020). To dig into the underlying relationship among mRNA, lncRNA, and miRNA post-ICH, a comprehensive ce-RNA network should be revealed.

**Figure 1:**
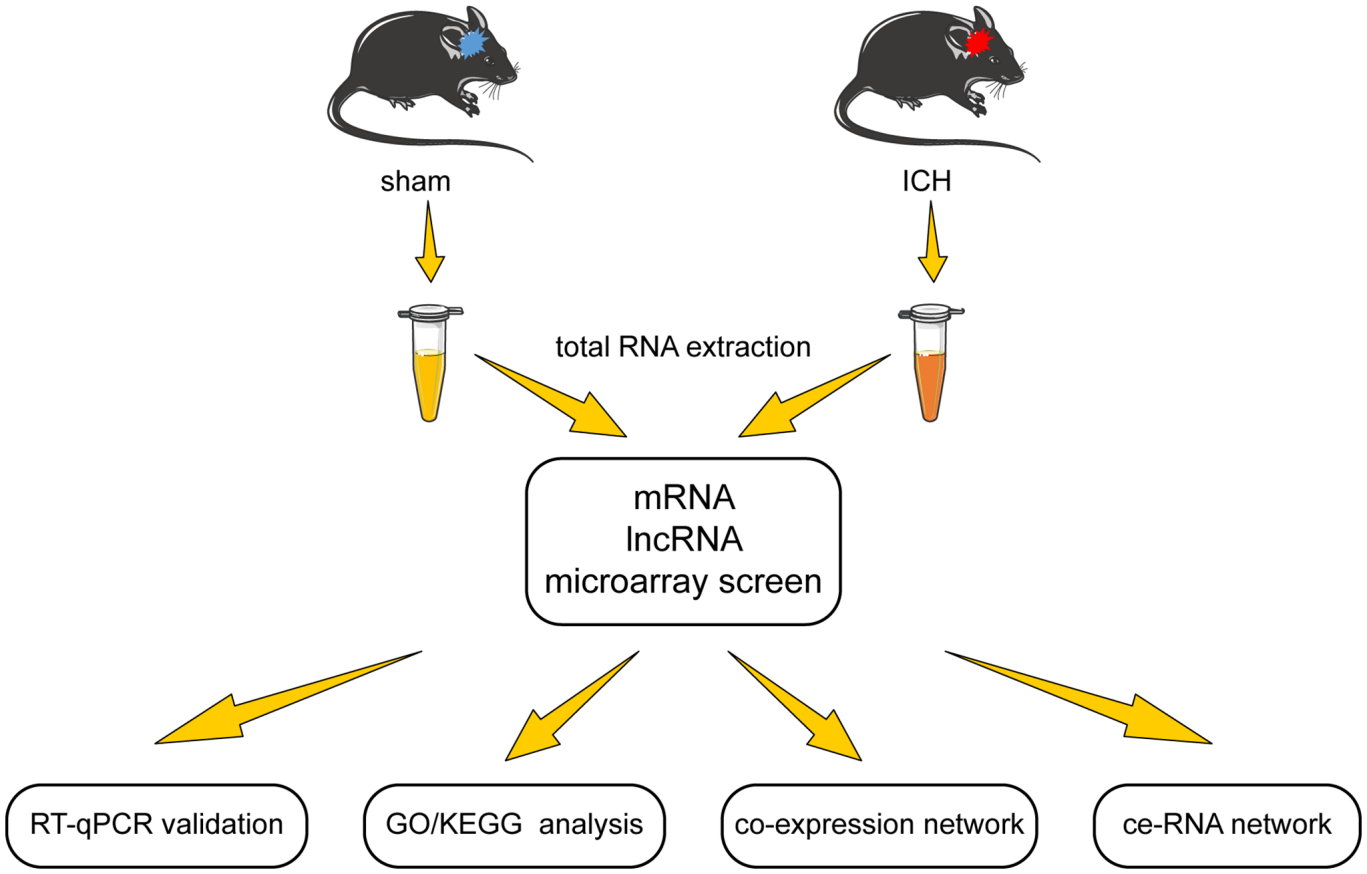
The flow chart of this study.

In this study, we examined the differentially expressed lncRNA and mRNA profiles in the acute phase of autologous blood-induced ICH animals. After validation of microarray results, we detected the functions of the differentially expressed mRNAs through GO/KEGG enrichment analysis. Furthermore, the lncRNA-mRNA co-expression network was established according to Pearson correlation coefficients. Moreover, to investigate the complicated correlation and interaction between lncRNA and mRNA, the ce-RNA network was carried out. The flow chart of this research is shown in Figure 1.

## 2. Materials and methods

### 2.1. Animals and ICH model

Studies were carried out on 8-week-old male C57BL/6 J mice obtained from the Laboratory Animal Centre of Central South University (CSU) (No. 2020sydw0929). All animals were housed in five per cage under stable environmental conditions (room temperature at approximately 25°C, relative humidity of the room at approximately 50%, 12 h light–dark cycle) with access to full water and food. All experimental procedures were approved by the Institutional Animal Care and Use Committee of CSU.

The ICH model was induced using autologous blood injection according to the published protocol (Rynkowski et al., 2008). Mice were randomly divided into sham and ICH groups. They were anesthetized by 0.3% pentobarbital sodium (22 mL/kg, i.p.). Autologous blood was obtained from the distal end of the mouse tail using a microinjector. For ICH induction, 15 μl fresh autologous blood was injected into the right globus pallidus at a rate of 1.5 μL min − 1. After injecting, the syringe was left in place for an additional 20 min to prevent backflow of autologous blood. The injection coordinates were 2 mm to the right, 0.5 mm posterior to Bregma, and 4 mm below the cortical surface. The sham group was injected with the same volume of 0.9% saline instead of autologous blood in the same site.

### 2.2. Behavioral tests

Behavioral tests were conducted on days 1, 3, and 7 after ICH induction. The modified neurological severity score (mNSS) and foot-fault test were used to evaluate the neurological injury (Li et al., 2019). In brief, mNSS assesses motor, sensory, and reflex abilities, and the foot-fault test evaluates the forelimbs dysfunction of mice. A higher score of mNSS indicates a more severe neurological function deficit. Meanwhile, data from the foot-fault test were presented as a percentage of foot faults (number of foot faults for left forelimb) per total number of steps.

### 2.3. Histological staining and quantify

The 3 to 4-μm coronal paraffin brain sections were stained following the instructions of the H&E (Servicebio, G1001&G1004) and Nissl’s staining kits (Servicebio, G1032), respectively. The cross-sectional hematoma areas were measured by ImageJ software in reference to a published study (Fei et al., 2022). To calculate the survival neurons, four peri-hematoma fields were photographed from each section at 200 magnifications. Within each filed, a fixed-size hematoma-free area was selected to count survival neurons. The number of survival neurons in the fixed-size area was calculated for statistical analysis.

### 2.4. mRNA and lncRNA microarray

Ten globus pallidus tissues (five from the sham group and five from the ICH group) were used to extract total RNA to detect microarray. The quantity and quality of total RNA were validated by NanoDrop ND-1000. Before microarray scanning, total RNA was purified by removing rRNAs (mRNA-ONLY™ Eukaryotic mRNA Isolation Kit, Epicenter) to obtain mRNAs. Next, the samples were amplified and transcribed into fluorescent cRNA (Arraystar Flash RNA Labeling Kit, Arraystar) and purified by RNeasy Mini Kit (Qiagen) subsequently. Arraystar Mouse lncRNA Microarray (V4.0, including about 22,692 mRNAs and 37,949 lncRNAs) was designed to profile mRNA along with lncRNA systematically. Agilent DNA Microarray Scanner (part number G2505C) was used for hybridized array scanning. The acquired array images were analyzed by Agilent Feature Extraction software (version 11.0.1.1). GeneSpring GX v12.1 software package (Agilent Technologies) performed quantile normalization and subsequent data processing. Differential expression of mRNAs and lncRNAs between the two groups were identified by applying a filter of fold change (FC) ≥ 2 and value of *p* <0.05.

### 2.5. LncRNA homology query

The sequence of lncRNA was obtained by referring to the NCBI and UCSC databases. Then, the sequence was aligned to the genome of human by the BLAT section of the UCSC database. The nucleotide blast tool of NCBI was used to validate the homology of lncRNA between mouse and human.

### 2.6. Real-time quantitative PCR

According to the manufacturer’s instructions, total RNA was extracted from globus pallidus tissue using a Trizol reagent (Takara, 9,108). Reverse transcription of mRNA/lncRNA used HiScript II Q RT SuperMix for qPCR (Vazyme, R223) as instructed by the manufacturer. Differential expression of mRNAs/lncRNAs was determined by RT-qPCR with ChamQTM Universal SYBR® qPCR Master Mix (Vazyme, Q711) following the manufacturer’s instructions, on a CFX Connect™ Real-time system (BIO-RAD, United States). Briefly, the cycling settings were as follows: incubation at 95°C for 5 min, followed by 40 cycles of 95°C for 10 s, 60°C for 30 s, and the instrument default melting curve acquisition procedures. GAPDH was used as the standardized control. Specific primers are listed in Table 1. The results of RT-qPCR were analyzed by the 2^−ΔΔCt^ method.

**Table 1:** RT-qPCR primers of candidate mRNAs and lncRNAs.

Name	Primers (forward and primer)
GAPDH	F: 5’ GGCAAATTCAACGGCACAGTCAAG 3′
	R: 5’ TCGCTCCTGGAAGATGGTGATGG 3′
Saa3	F: 5’ AGCTGACCAGTTTGCCAATGAGTG 3′
	R: 5’ ACCCAGTAGTTGCTCCTCTTCTCG 3′
Col10a1	F: 5’ ATGCCGCTTGTCAGTGCTAACC 3′
	R: 5’ GGGTCGTAATGCTGCTGCCTATTG 3′
Mmp13	F: 5’ ACAGTTGACAGGCTCCGAGAAATG 3’
	R: 5’ CCACATCAGGCACTCCACATCTTG 3’
Oxt	F: 5’ ACCATCACCTACAGCGGATCTCAG 3’
	F: 5’ GTCAGAGCCAGTAAGCCAAGCAG 3’
Pchhb6	F: 5’ GCAGCACGACTCTTCACAGGATG 3’
	R: 5’ CGAACAGCAGCACAGACAGGAG 3’
Resp18	F: 5’ GTGAGGAGCAAGCAAGAGGAGAAAC 3’
	R: 5’ GTGGTAAAGCATCGGTCCCTGTTC 3’
ENSMUST00000161503	F: 5’ TCTCAACTGCCCTTCACTCTCTTAG 3’
	R: 5’ CATCCTTACCTCTCCTCCACTTCC 3’
AK041738	F: 5’ TCTCTTATCTGCCCCTGGACTTGTG 3’
	R: 5’ CAAACCACTCACTCCCACTTCCTG 3’
ENSMUST00000210375	F: 5’ GCCACAACCAGCGCAGAC 3’
	R: 5’ TCCTAGTCGTGCTTGAGAGTGAG 3’
ENSMUST00000126225	F: 5’ TGACCTCCCGGATGTTCTCT 3’
	R:5’ TTTGTTTTGCAGGTCACGGC 3’
ENSMUST00000152254	F: 5’ GGTGGAAACATCAGGGCAGAAG 3’
	R: 5’ GTTGGGAAGAAGGTCACTAGGAATG 3’
AF177020	F: 5’ AGTAGCCTAGTCTAGCACCCAACC 3’
	R: 5’ CTGAGGGCGTGTTCTGACTTATAGC 3’

### 2.7. Go and KEGG analysis

The roles differentially expressed mRNAs played in biological pathways were applied by GO and KEGG analysis. Metascape database was used to perform GO (biological process, cellular component, molecular function) and KEGG-enriched pathways (Zhou et al., 2019).

### 2.8. Establishment of lncRNA-mRNA co-expression network

Co-expression analysis was performed using the Sangerbox tools,^1^ which is a free online platform for data analysis. Pearson correlation coefficients (PCCs) were calculated to present the correlation between differentially expressed mRNAs and lncRNAs. LncRNA-mRNA pairs of co-expression were defined by |PCCs| > 0.99 and value of *p* <0.05. Cytoscape (version v3.9.1) was used for visualization of the co-expression network.

**Table 2:** Top 5 up-regulated and down-regulated mRNAs post-ICH.

Transcription ID	Gene symbol	Fold change (FC)	value of *p*	Regulation
NM_011315	Saa3	168.1430479	0.000232152	up
NM_009925	Col10a1	86.4408001	0.000152025	up
NM_009690	Cd5l	51.5293354	7.95593E-05	up
NM_008607	Mmp13	43.8708593	0.000143571	up
NM_008599	Cxcl9	31.6133097	0.000279797	up
NM_001123362	Prdm12	5.1310422	0.03269006	down
NM_011025	Oxt	2.6697481	0.042354851	down
NM_013833	Rax	2.5607242	0.008107894	down
NM_009049	Resp18	2.5185397	0.023800052	down
NM_153079	Nmur2	2.4707521	0.027802601	down

### 2.9. Construction of ce-RNA network

We selected DIANALncBaseV.3^2^ to establish the lncRNA-miRNA interaction linkage. mRNA-miRNA interaction was predicted through miRDB-MicroRNA Target Prediction Database^3^ by setting the screening target score over 80. Then, the data of lncRNA-miRNA and mRNA-miRNA were integrated. Cytoscape (version v3.9.1) was used for visualization of the ce-RNA network. MCODE plugin of Cytoscape screened hub clusters according to filter criteria (degree cutoff: 0.2, node score cutoff: 0.2, k-core: 2, and max. Depth: 100).

### 2.10. Statistical analysis

GraphPad Prism 9 (GraphPad Software, Inc., La Jolla, CA, United States) was used for statistical analysis. Statistical differences were analyzed by t-test. Data from behavioral tests were analyzed by a two-way ANOVA test followed by Bonferroni’s multiple comparisons. The results were expressed as the mean ± SEM. A value of *p* of <0.05 was defined as statistically significant.

## 3. Results

### 3.1. Neurological deficits and histological alteration post-ICH

**Figure 2:**
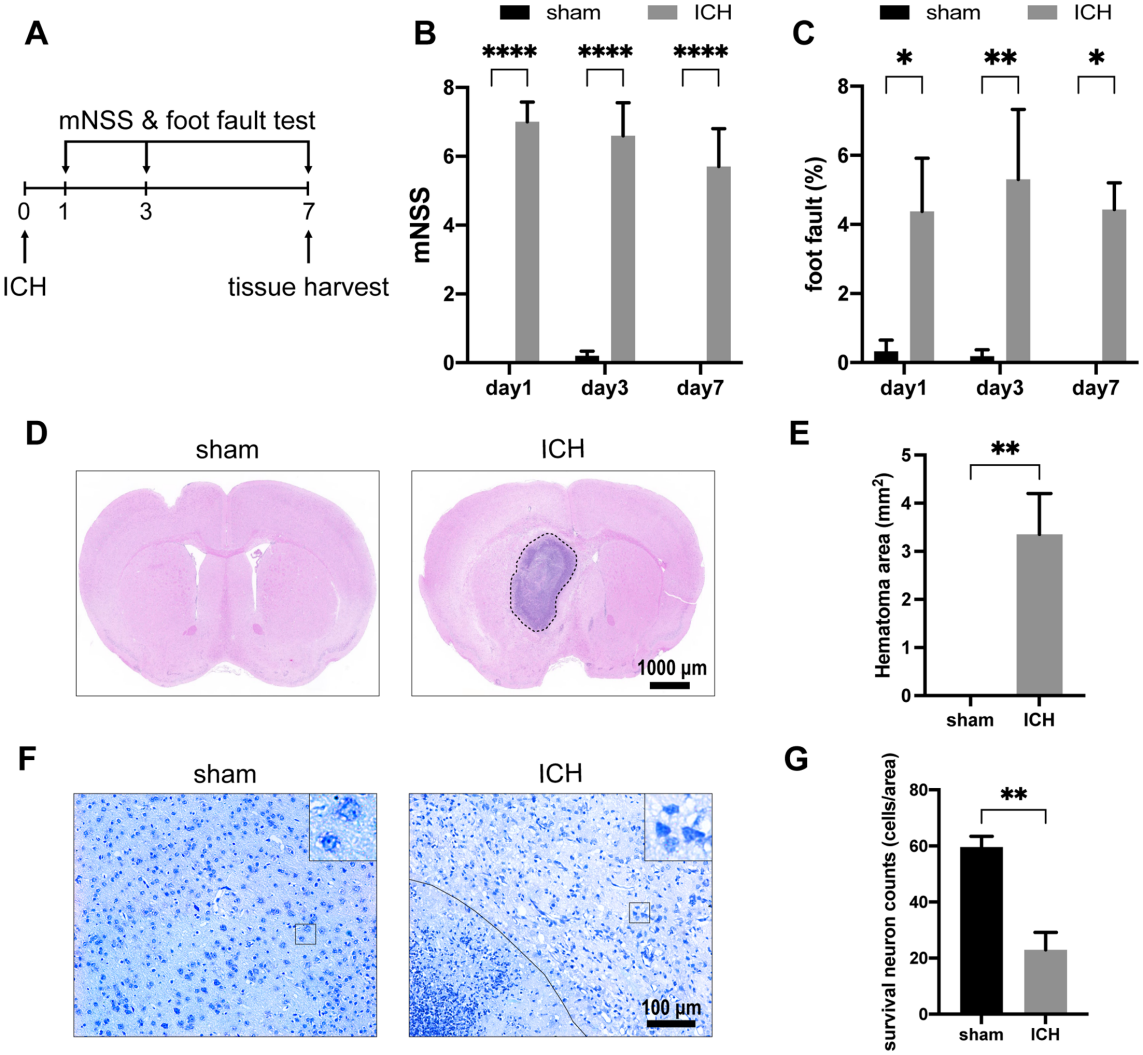
ICH-induced neurological deficits and histology alterations. **(A)** Schedule of behavioral tests and tissue harvest. The mNSS **(B)** and foot-fault test **(C)** were measured for the two groups at three time points (day 1, day 3, day 7) post-ICH. (*n* = 7–10 mice in each group) Representative H&E staining **(D)** and Nissl staining **(F)** images on day 7 post-ICH. **(E)** Compared with the sham group, the ICH group exists significant hematoma. (*n* = 5 mice for each group) **(G)** The number of survival neurons in the ICH group was significantly decreased compared with that in the sham group. (*n* = 4 mice for each group) Data are presented as mean ± SEM, ^*^p<0.05, ^**^p<0.01, ^****^p<0.001.

To evaluate the alteration of neurological function of mice after ICH, mNSS and foot-fault rates were recorded on days 1, 3, and 7 after the operation (*n* = 7–10) (Figure 2A). On the first day after ICH, the mNSS scores and foot-fault rates significantly increased compared with the sham group (Figures 2B,C). These neurological deficits persisted until day 7 when the tissues were harvested. Histological staining was conducted on brain tissues obtained on day 7 post-ICH. H&E staining revealed the hematoma area after stereotaxic injected autologous blood at the globus pallidus on day 7 (*n* = 5) (Figures 2D,E). In Nissl’s staining, injured neurons show up as dark neurons characterized by massive shrinkage and abnormal basophilia (Ooigawa et al., 2006). Therefore, we used the number of survival neurons to reflect the severity of neurological damage. The number of survival neurons decreased notably in contrast to dark neurons after ICH (*n* = 4) (Figures 2F,G).

### 3.2. Differential expression of mRNAs and lncRNAs in ICH

**Figure 3:**
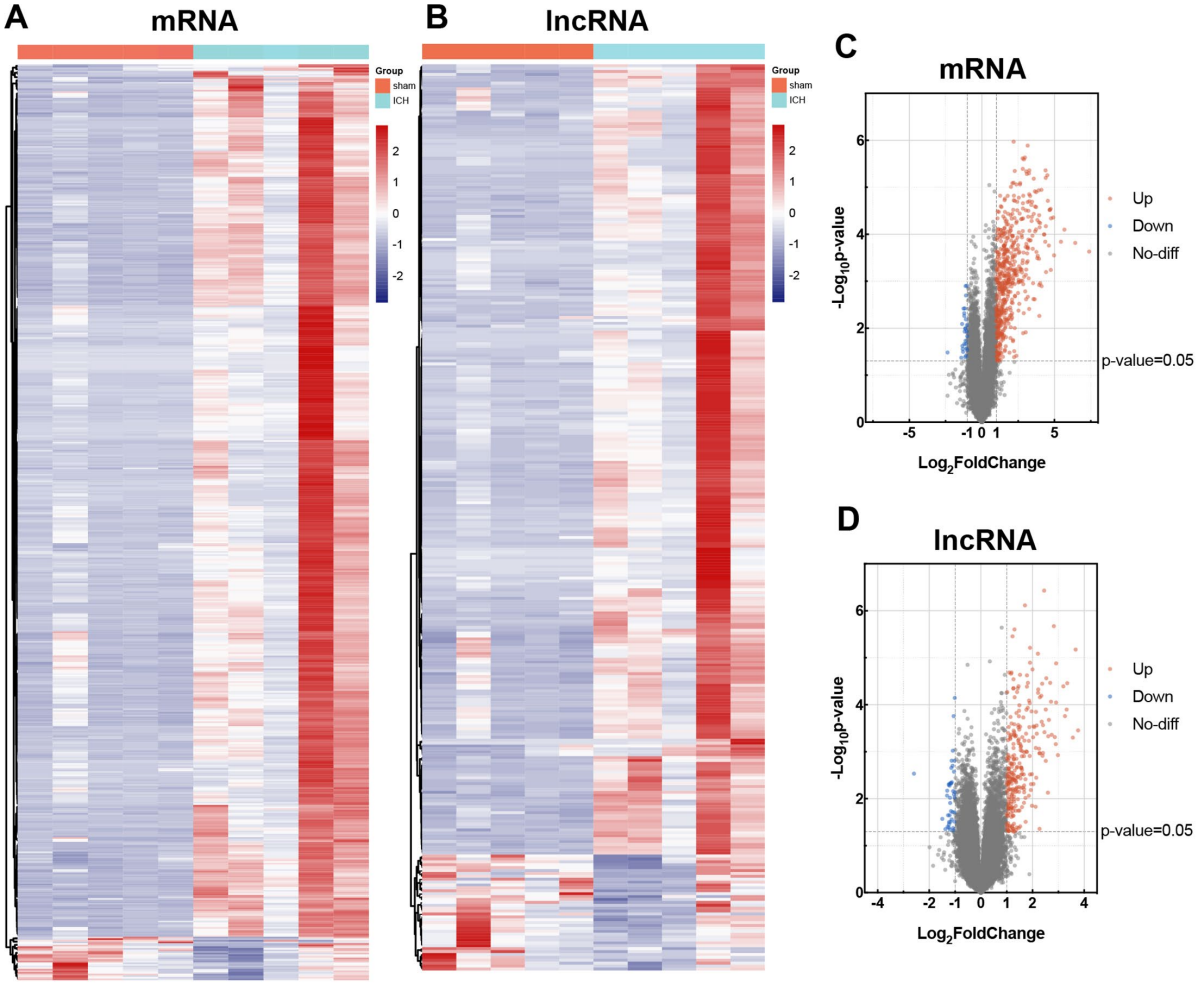
Differential expression of mRNAs and lncRNAs in sham and ICH group. Heat maps of differentially expressed mRNAs **(A)** and lncRNAs **(B)**. Cluster analysis was carried out with rows. Volcano maps of differentially expressed mRNAs **(C)** and lncRNAs **(D)**. Differentially expressed mRNAs and lncRNAs were according to the cutoff of log |FC| ≥ 2 and value of *p* <0.05. The up-regulated transcripts were marked in red, and the down-regulated transcripts in blue.

In order to investigate the alteration of mRNAs and lncRNAs in ICH, we used Arraystar Mouse lncRNA Microarray, including 22,692 mRNAs and 37,949 lncRNAs. Before microarray scanning, the quantity and quality of RNAs and labeling efficiency were validated by NanoDrop ND-1000 and specific activity (Supplementary Tables S1, S2). With an FC ≥ 2 and value of *p* <0.05 filtering, heat maps and volcano maps for differentially expressed mRNAs and lncRNAs were achieved (Figure 3). Five hundred and seventy mRNAs (including 543 up-regulated transcripts and 27 down-regulated transcripts) and 313 lncRNAs (including 273 up-regulated transcripts and 40 down-regulated transcripts) were identified between sham and ICH groups (*n* = 5). The heatmaps were also performed to visualize the cluster analysis of differentially expressed mRNAs and lncRNAs (Figures 3A,B). The top 5 up/down-regulated mRNAs and lncRNAs were listed in Tables 2, 3.

**Table 3:** Top 5 up-regulated and down-regulated lncRNAs post-ICH.

GeneID	Gene symbol	Fold change (FC)	*p*-value	Regulation
ENSMUSG00000085786	Gm15987	13.6022041	0.000351216	up
AK143011	AK143011	12.7551382	6.7097E-06	up
ENSMUSG00000075010	AW112010	11.819704	0.000498888	up
ENSMUSG00000047415	Gpr68	10.1714873	3.46477E-05	up
ENSMUSG00000023349	Clec4n	10.0060345	0.000126743	up
AK030988	AK030988	6.0191623	0.002926642	down
ENSMUSG00000097620	4921514A10Rik	2.8131091	0.026730509	down
ENSMUSG00000087694	A530058N18Rik	2.5930208	0.044473003	down
AF177020	AF177020	2.4809815	0.008580349	down
ENSMUSG00000099696	2900052N01Rik	2.4651633	0.006742548	down

**Table 4:** Top 5 GO terms enriched by differentially expressed mRNA post-ICH.

GO	Category	Description	Count	Log10(P)
GO:0008009	MF	Chemokine activity	14	−12.03
GO:0038187	MF	Pattern recognition receptor activity	11	−9.96
GO:0042287	MF	MHC protein binding	14	−9.43
GO:0004857	MF	Enzyme inhibitor activity	35	−9.12
GO:0140375	MF	Immune receptor activity	19	−8.63
GO:0032103	BP	Positive regulation of response to external stimulus	75	−37.57
GO:0006954	BP	Inflammatory response	74	−33.6
GO:0002683	BP	Negative regulation of immune system process	62	−26.76
GO:0050778	BP	Positive regulation of immune response	75	−26.49
GO:0001775	BP	Cell activation	69	−21.4
GO:0009897	CC	External side of plasma membrane	69	−22.64
GO:0000323	CC	Lytic vacuole	51	−14.43
GO:0045121	CC	Membrane raft	34	−9.05
GO:0098797	CC	Plasma membrane protein complex	41	−7.88
GO:0005581	CC	Collagen trimer	12	−5.95

**Figure 4:**
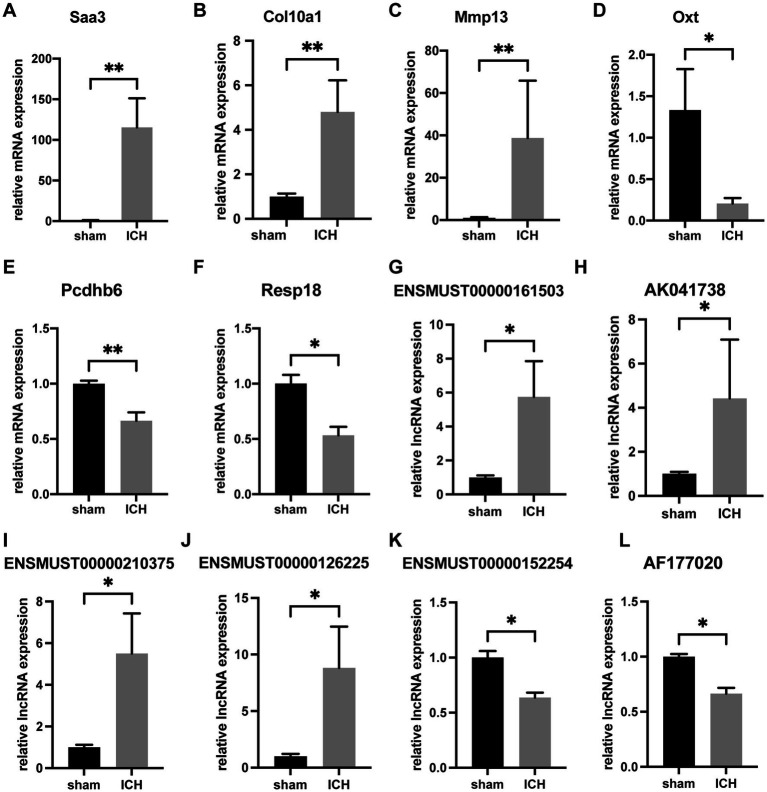
RT-qPCR confirmation of differentially expressed mRNAs and lncRNAs. The relative expression of Saa3 **(A)**, Col10a1 **(B)**, Mmp13 **(C)**, ENSMUST00000161503 **(G)**, AK041738 **(H)**, ENSMUST00000210375 **(I)**, and ENSMUST00000126225 **(J)** was upregulated in ICH group. The relative expression of Oxt **(D)**, Pcdhb6 **(E)**, Resp18 **(F)**, ENSMUST00000152254 **(K)**, and AF177020 **(L)** was downregulated in the ICH group. (*n* = 4–5 mice in each group) Data are presented as mean ± SEM, *p<0.05, **p<0.01.

To verify the accuracy of microarray, 6 mRNAs (Saa3, Col10a1, Mmp13, Oxt, Pcdhb6 and Resp18) and 6 lncRNAs (ENSMUST00000161503, AK041738, ENSMUST00000210375, ENSMUST00000126225, ENSMUST00000152254, and AF177020) were selected for RT-qPCR. The 6 validated lncRNAs were confirmed a curtained conservation with human (Supplementary Table S3). Consistent with the results of array scanning, RT-qPCR confirmed that the expressions of Saa3, Col10a1, Mmp13, ENSMUST00000161503, AK041738, ENSMUST00000210375, and ENSMUST00000126225 were up-regulated post-ICH (*n* = 4–5) (Figures 4A–C,G). Meanwhile, the expressions of Oxt, Pcdhb6, Resp18, AK030988, ENSMUST00000152254, and AF177020 were lower in the ICH group than that of the sham group (*n* = 4–5) (Figures 4D–F,K). The RT-qPCR results supported the reliability of the microarray.

### 3.3. Functional enrichment of differentially expressed mRNAs

**Figure 5:**
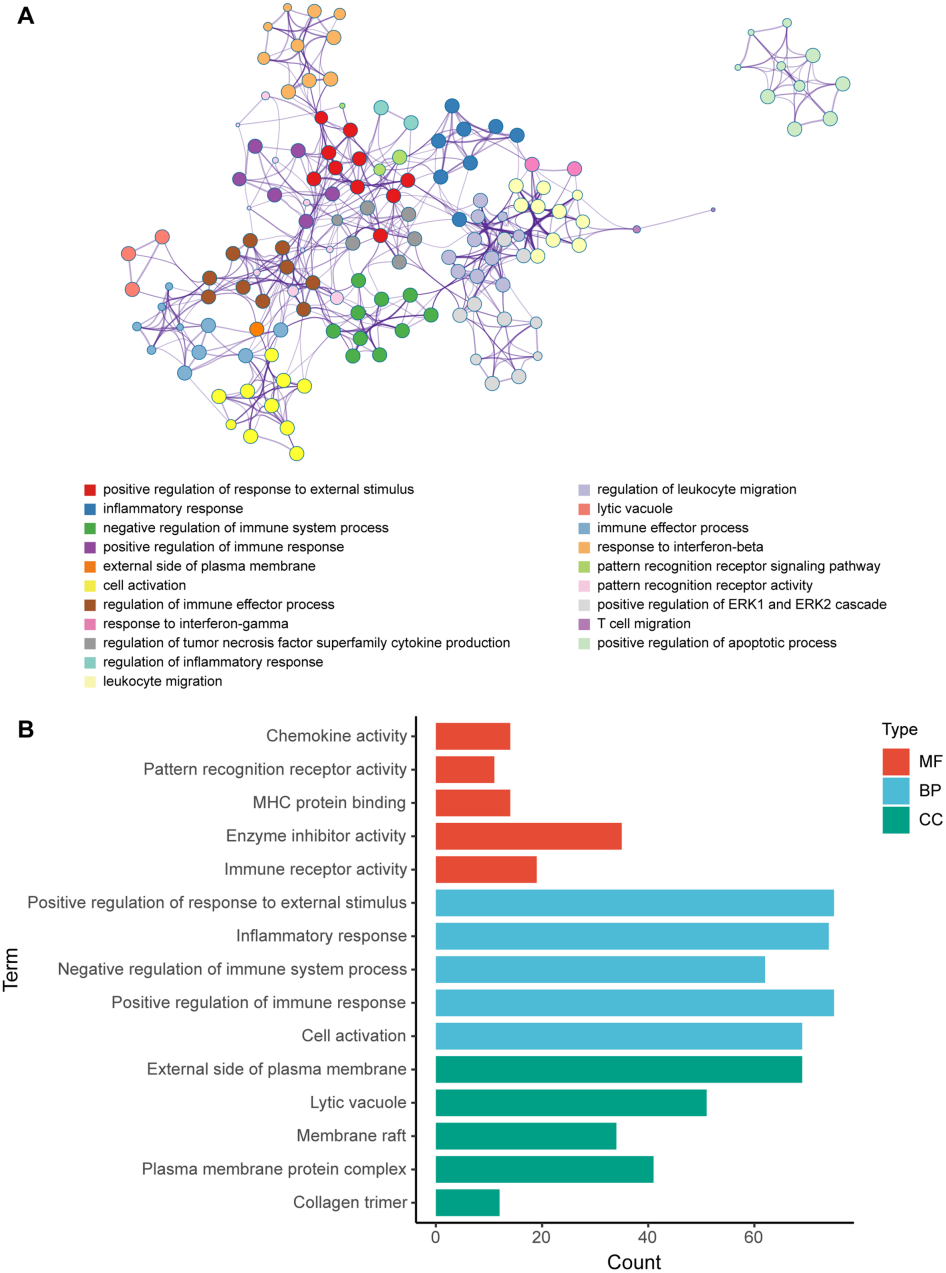
GO enrichment analysis of differentially expressed mRNA post-ICH. **(A)** Network of enriched GO terms. The nodes were colored by cluster-ID. If similarity >0.3, the nodes were connected by edges. **(B)** The bar graph of the top 5 GO terms in BP, CC, and MF, respectively.

Metascape database was used to analyze the functions of 570 differentially expressed mRNAs. GO analysis was performed in three dimensions involving biological process (BP), cellular compound (CC), and molecular function (MF). In total, 1,046 GO items were enriched, including 856 BP terms, 76 CC terms, and 114 MF terms. A subset of enriched terms was rendered as a network plot and colored by cluster-ID. Simultaneously, the plots (with a similarity >0.3) were connected by edges. Finally, we selected 20 clusters of best value of ps to be visualized by the network (Figure 5A). The bar graph showed the top 5 terms in BP, CC, and MF, respectively (Figure 5B) and listed in Table 4. BP was mainly involved in chemokine activity, pattern recognition, MHC protein binding, enzyme inhibitor activity, and immune receptor. CC was mainly focused on the positive regulation of response to external stimulus, inflammatory response, negative regulation of immune system process, positive regulation of immune response, and cell activation. Meanwhile, MF was enriched in the external side of plasma membrane, lytic vacuole, membrane raft, plasma membrane protein complex, and collagen trimer.

**Figure 6:**
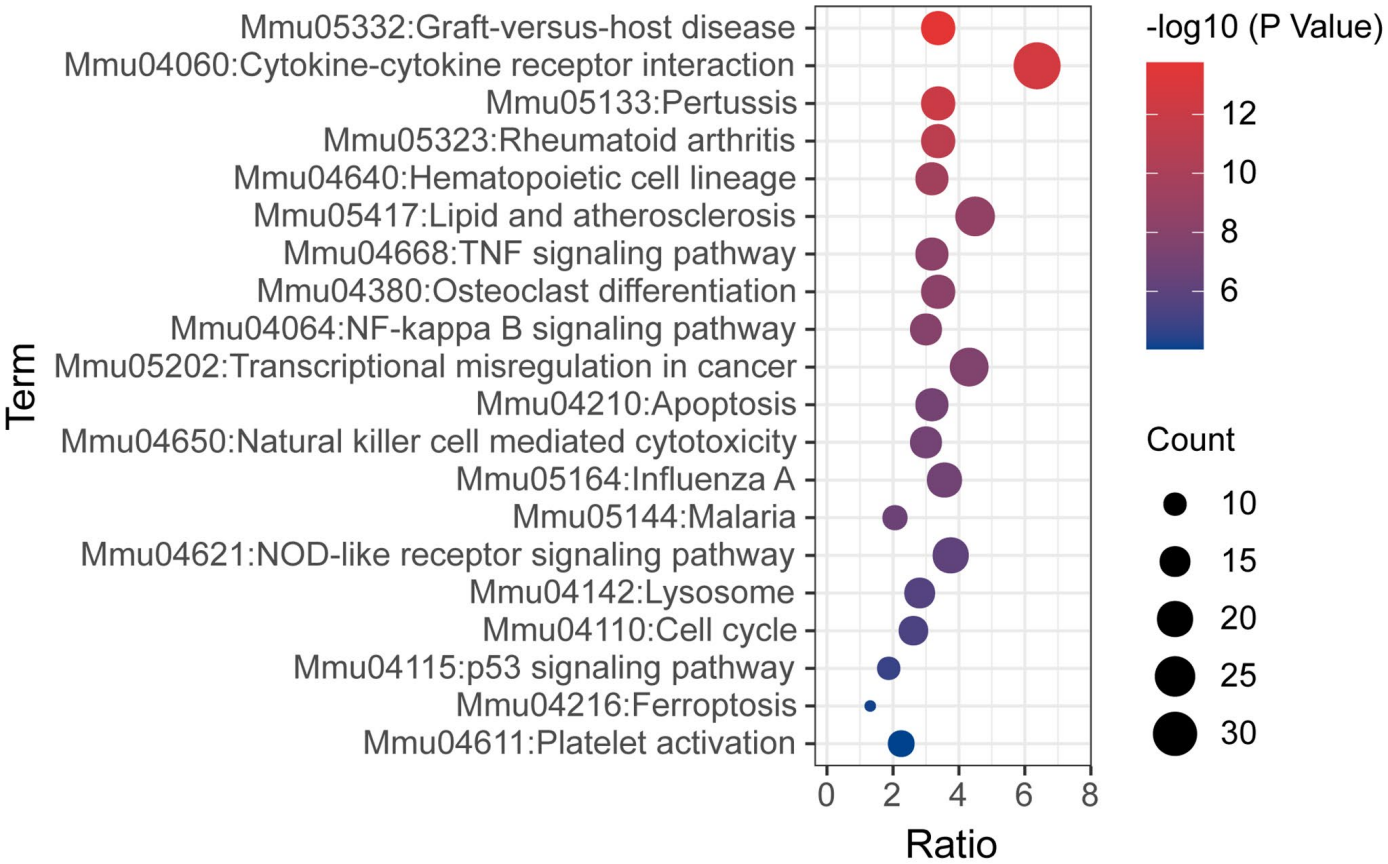
Bubble graph representing KEGG pathways from the enrichment analysis of differentially expressed mRNA post-ICH. The top 20 enriched terms were shown ranked by value of *p*, and the size of bubbles represents the gene counts corresponding to the pathway.

KEGG pathway analysis was also conducted based on the Metascape platform. There were 78 pathways enriched with differentially expressed mRNAs. Ranked by lg value of *p* from the smallest to the largest, the top 20 pathways were shown in a bubble graph (Figure 6) and listed in Table 5, mainly containing cytokine-cytokine receptor interaction, hematopoietic cell lineage, TNF signaling pathway, NF-κB signaling pathway, apoptosis, natural killer cell mediated cytotoxicity, NOD-like receptor signaling pathway, lysosome, p53 signaling pathway, ferroptosis, and platelet activation.

**Table 5:** Top 20 KEGG terms enriched by differentially expressed mRNA post-ICH.

GO	Description	Count	Log10(*p*)	Log10(*q*)
mmu05332	Graft-versus-host disease	18	−13.75	−11.21
mmu04060	Cytokine-cytokine receptor interaction	34	−12.62	−10.78
mmu05133	Pertussis	18	−12.09	−10.41
mmu05323	Rheumatoid arthritis	18	−11.13	−9.62
mmu04640	Hematopoietic cell lineage	17	−9.57	−8.31
mmu05417	Lipid and atherosclerosis	24	−8.66	−7.43
mmu04668	TNF signaling pathway	17	−8.29	−7.12
mmu04380	Osteoclast differentiation	18	−8.26	−7.12
mmu04064	NF-kappa B signaling pathway	16	−7.92	−6.85
mmu05202	Transcriptional misregulation in cancer	23	−7.67	−6.63
mmu04210	Apoptosis	17	−7.06	−6.03
mmu04650	Natural killer cell mediated cytotoxicity	16	−7.03	−6.01
mmu05164	Influenza A	19	−6.9	−5.9
mmu05144	Malaria	11	−6.71	−5.73
mmu04621	NOD-like receptor signaling pathway	20	−6.12	−5.2
mmu04142	Lysosome	15	−5.64	−4.75
mmu04110	Cell cycle	14	−5.35	−4.48
mmu04115	p53 signaling pathway	10	−4.8	−3.94
mmu04216	Ferroptosis	7	−4.18	−3.34
mmu04611	Platelet activation	12	−4.04	−3.23

### 3.4. LncRNA-mRNA co-expression network

**Figure 7:**
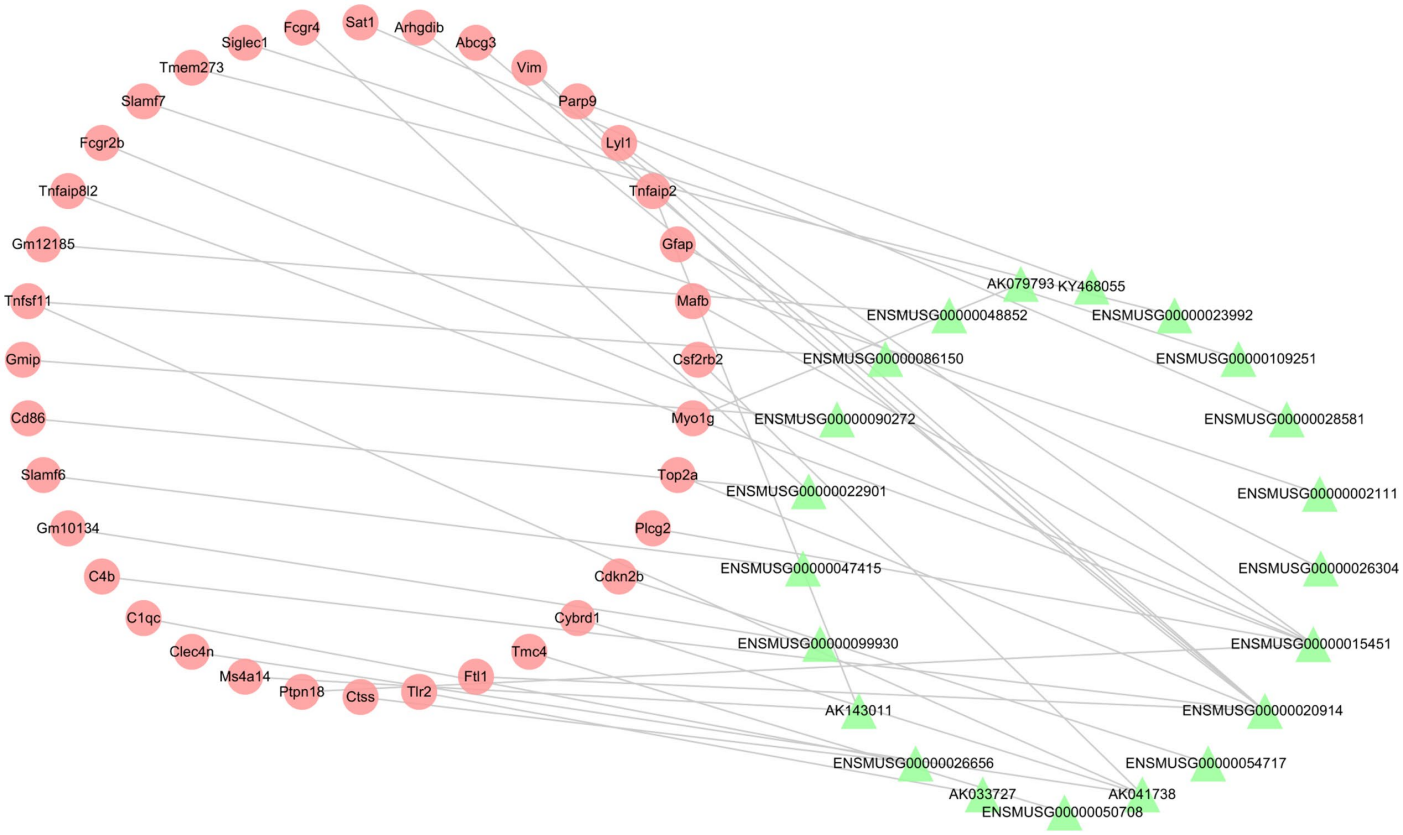
The lncRNA-mRNA co-expression network post-ICH. The red circles represent mRNAs, and the green triangles represent lncRNAs.

To investigate the relationship between differentially expressed lncRNAs and mRNAs, the Pearson correlation coefficients were calculated. If the absolute value of PCCs >0.99 and the value of *p* <0.05, we defined the lncRNA-mRNA pairs as co-expression. The co-expression network contained 57 nodes (21 lncRNAs and 36 mRNAs) and 38 connections (Figure 7). The top 10 co-expression lncRNA-mRNA pairs were ENSMUSG00000015451-Tnfaip8l2, ENSMUSG00000099930-Gm10134, ENSMUSG00000026656-Ctss, ENSMUSG00000020914-Top2a, KY468055-Parp9, ENSMUSG00000023992-Tmem273, ENSMUSG00000090272-Gmip, AK143011-Ms4a14, AK041738-Cybrd1 and AK041738-Csf2rb2 (Table 6).

**Table 6:** LncRNA-mRNA co-expression pairs. (|PCCs| > 0.99).

lncRNA	mRNA	*p*-value	PCCs
ENSMUSG00000015451	Tnfaip8l2	1.92E-10	0.997425788
ENSMUSG00000099930	Gm10134	2.23E-10	0.997327088
ENSMUSG00000026656	Ctss	8.71E-10	0.996239743
ENSMUSG00000020914	Top2a	2.23E-09	0.99523903
KY468055	Parp9	4.14E-09	0.994444226
ENSMUSG00000023992	Tmem273	4.54E-09	0.994315768
ENSMUSG00000090272	Gmip	4.86E-09	0.994217239
AK143011	Ms4a14	5.96E-09	0.993914269
AK041738	Cybrd1	7.00E-09	0.993664268
AK041738	Csf2rb2	7.04E-09	0.993654819
ENSMUSG00000020914	Vim	9.16E-09	0.993221134
ENSMUSG00000002111	Slamf7	1.01E-08	0.99305264
ENSMUSG00000022901	Cd86	1.47E-08	0.992374707
ENSMUSG00000020914	Vim	1.47E-08	0.992362848
ENSMUSG00000015451	Ptpn18	1.62E-08	0.992180803
ENSMUSG00000054717	Cdkn2b	1.68E-08	0.992106452
ENSMUSG00000015451	Mafb	1.72E-08	0.992057591
ENSMUSG00000028581	Sat1	1.93E-08	0.991830154
ENSMUSG00000015451	Fcgr2b	1.94E-08	0.991814958
ENSMUSG00000047415	Slamf6	2.04E-08	0.99171702
ENSMUSG00000026656	C1qc	2.27E-08	0.991492486
ENSMUSG00000050708	Tmc4	2.29E-08	0.991472954
ENSMUSG00000026304	Gfap	2.36E-08	0.991409206
ENSMUSG00000020914	Abcg3	2.52E-08	0.991267119
ENSMUSG00000022901	Fcgr4	2.54E-08	0.991245292
ENSMUSG00000048852	Gm12185	2.68E-08	0.991130117
ENSMUSG00000020914	C4b	2.90E-08	0.990951017
AK033727	Tlr2	2.94E-08	0.990920626
AK079793	Myo1g	2.94E-08	0.990920012
ENSMUSG00000015451	Lyl1	3.01E-08	0.9908701
ENSMUSG00000109251	Siglec1	3.35E-08	0.990620722
ENSMUSG00000086150	Tnfsf11	3.50E-08	0.990514182
AK041738	Tnfsf11	3.57E-08	0.990467798
AK041738	Clec4n	3.84E-08	0.990295043
ENSMUSG00000020914	Ftl1	3.93E-08	0.990235332
ENSMUSG00000015451	Plcg2	4.11E-08	0.990127434
AK143011	Tnfaip2	4.20E-08	0.990072078
ENSMUSG00000020914	Arhgdib	4.25E-08	0.990042744

### 3.5. LncRNA-miRNA-mRNA (ce-RNA) network

**Figure 8:**
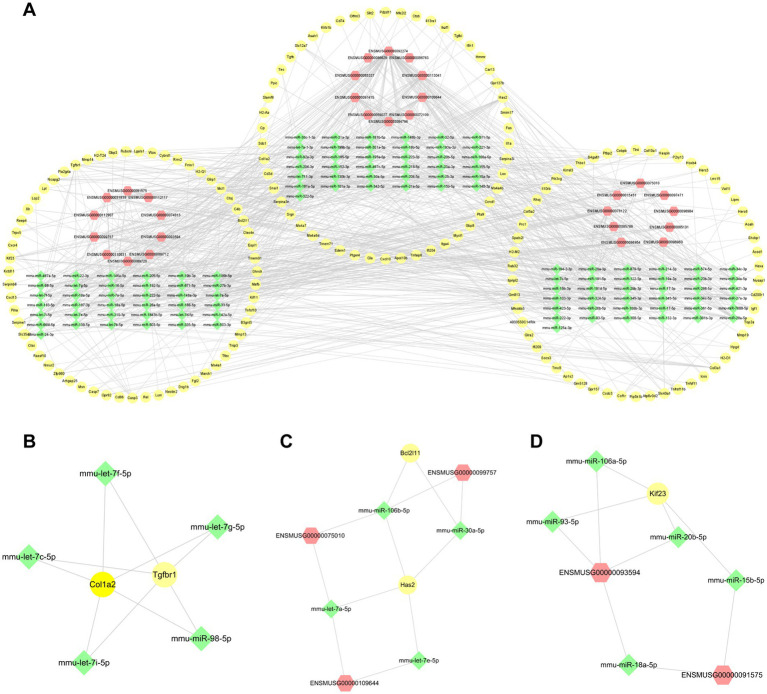
The lncRNA-miRNA-mRNA interactions post-ICH. **(A)** The ce-RNA network post-ICH. The hub clusters of the ce-RNA network: are Cluster1 **(B)**, Cluster2 **(C)**, and Cluster3 **(D)**. The yellow circles represent mRNAs, green rhombuses represent miRNAs, and red hexagons represent lncRNAs.

For the purpose of exploring further functions based on the mechanism of competitive endogenous RNA (ce-RNA), we predicted the miRNAs which were correlated with differentially expressed lncRNAs and mRNAs. We defined lncRNA-miRNA pairs and mRNA-miRNA pairs with target scores >80 as closely correlated. A total of 570 differentially expressed mRNAs were correlated with 1,514 miRNAs. Additionally, 242 miRNAs were predicted by 313 differentially expressed lncRNAs based on the DIANALncBaseV.3 database. Totally, we defined 490 mRNA-miRNA pairs and 416 lncRNA-miRNA pairs. We combined with miRNAs predicted by mRNAs and lncRNAs to establish the ce-RNA network (Figure 8A). The ce-RNA network was including 303 nodes (29 lncRNAs, 163 mRNAs, and 111 miRNAs) and 906 edges. Then, we used MCODE, a plugin module of Cytoscape, to analyze the ce-RNA network. Three clusters were screened based on the filter criteria (degree cutoff: 0.2, node score cutoff: 0.2, k-core: 2, and max. Depth: 100) (Figures 8B–D). Cluster1 was composed with 2 mRNAs (Col1a2 and Tgfbr1) and 5 mircoRNAs (mmu-let-7f-5p, mmu-let-7c-5p, mmu-let-7i-5p, mmu-let-7 g-5p and mmu-miR-98-5p) (Figure 8B). In Cluster2 (Has2, Bcl2l11, ENSMUSG00000099757, ENSMUSG00000075010, ENSMUSG00000109644, mmu-miR-106b-5p, mmu-miR-30a-5p, mmu-let-7a-5p, mmu-let-7e-5p) (Figure 8C), there were 7 mRNA-miRNA-lncRNA pairs be screened comparing with 4 pairs in Cluster3 (Kif23, ENSMUSG00000093594, ENSMUSG00000091575, mmu-miR-106a-5p, mmu-miR-93-5p, mmu-miR-20b-5p, mmu-miR-15b-5p, mmu-miR-18a-5p) (Figure 8D).

## 4. Discussion

In our previous study, we investigated the relationship between mRNA and lncRNA in the ICH model 21 days (chronic phase) post-ICH (Hanjin et al., 2018). In this study, to understand the pathophysiological changes during acute and subacute phases of intracerebral hemorrhage, we used microarray to obtain the differential expression of mRNAs and lncRNAs between sham and ICH groups. Afterward, we validated the accuracy of results from the microarray relying on RT-qPCR. The relative expression of 3 mRNAs and 4 lncRNAs (Saa3, Col10a1, Mmp13, ENSMUST00000161503, AK041738, ENSMUST00000210375, and ENSMUST00000126225) were up-regulated in contrast to the other 3 mRNAs and 2 lncRNAs (Oxt, Pcdhb6, Resp18, ENSMUST00000152254 and AF177020). These were consistent with the results of microarray, demonstrating that the results of microarray were reliable. To investigate the hiding function of differentially expressed mRNAs and lncRNAs, GO/KEGG was performed. The pathways and functions enriched by GO/KEGG mainly focused on the pathophysiological processes related to immune response, inflammatory factors, inflammatory cell activation, and apoptosis. In addition, the results of GO/KEGG were also involved in ferroptosis, platelet activation, and biological processes of membrane. The regulations of mRNA transcription and translation by lncRNA have attracted increasing attention. Besides, we are also concerned about the ternary regulatory relationship of mRNA-miRNA-lncRNA. Therefore, to gain further insight into the association between differentially expressed mRNAs and lncRNAs, we constructed the lncRNA-mRNA co-expression network and the ce-RNA network. Thirty-eight lncRNA-mRNA pairs were focused on in the co-expression network. Three hub clusters were selected from the complicated ce-RNA network.

In our study, the top five up-regulated mRNAs were Saa3, Col10a1, Cd5l, Mmp13, and Cxcl19. To date, none of these five mRNAs have been investigated in ICH. Saa3 is the target gene of C/EBPβ, which is up-regulated in cerebral ischemia (Hanjin et al., 2018). However, the exact function of Saa3 in stroke is still unclear. There is a study speculates that Saa3 plays a role in the recruitment and adhesion of monocytes (Han et al., 2007). Under the hypoxic condition, Col10a1 was up-regulated in certain types of cells, including human retinal microvascular endothelial cells, umbilical vein endothelial cells, RPE19 cells, and RF/6A cells (Lv et al., 2022). It indicates that Col10a1 may be related to microvascular formation and angiogenesis. Danger-associated molecular pattern (DAMP) is a new phase secondary to the primary injury, such as cell death. The sterile inflammation occurs following DAMP. It was found that CD5 antigen-like (Cd5l) administration attenuates sterile inflammation and that Cd5l deficiency courses severe neurological damage with higher mortality (Maehara et al., 2021). The up-regulation of Cd5l and Cxcl19 in the study may be a compensatory elevation after ICH. As a family of diverse zinc endopeptidases, matrix metalloproteinases (Mmps) play a major role in the physiology and pathophysiology of the central nervous system (CNS) (Rosell and Lo, 2008). The baseline of plasma Mmp13 has been proven to be strongly associated with the severity of the ischemic stroke and indicates poorer outcomes (Ma et al., 2016). Similar to the top 5 up-regulated mRNAs, most of the top 5 down-regulated mRNAs are still uninvestigated in ICH. Prdm12 was confirmed to contribute to nervous system development (Kinameri et al., 2008). Oxytocin (OXT) is the ligand of the Oxytocin receptor (OXTR), which is located in microvascular endothelial cells. Exogenous OXT administration benefits cerebral ischemia by reducing ROS and neuron apoptosis (Stary et al., 2019). Rax also plays a significant role in forebrain development and neuronal regeneration (Muranishi et al., 2012; Stary et al., 2019). Moreover, Resp18 knockdown mice have poorer locomotor activity in Parkinson’s disease (Su et al., 2018). It may prompt Resp18 to be involved in CNS function. Nmur2 (neuromedin U receptor 2), the receptor of neuromedin U (NMU), is identified predominantly centrally. The role of receptor-ligand binding mainly involves central energy homeostasis, thermoregulation, circadian rhythms, and stress response (Malendowicz and Rucinski, 2021). In brief, the top five up-regulated mRNAs are mainly associated with inflammation and angiogenesis. While most of the top 5 down-regulated mRNAs are related to CNS development, neuroprotection, and neurodegeneration. The specific roles of these genes in the pathophysiological process of ICH remain to be further investigated.

The essential biological function and pathways enriched by GO/KEGG are immune responses and inflammation. The CNS injury after ICH includes primary injury and secondary injury. The extravasated blood from ruptured vessels causes hematoma formation within a few hours (Tschoe et al., 2020). The resident glial cells are suggested to be the earliest inflammatory cells in response to the primary injury. Additionally, neuroinflammation is a significant progress of secondary injury. Neuroinflammation is triggered by thrombin, Hemoglobin (Hb), Hemin, iron, and other stimulator released by blood and dead cells (Zhou et al., 2014). Besides, brain edema aggravates damage to neurons and glial cells, causing complex inflammatory cascades. Therefore, inflammation and immune response extend almost through the entire process of ICH. Notably, biological processes of GO analysis enriched for two opposing signaling pathways simultaneously, negative regulation of immune system progress and positive regulation of immune response. Activation of immune response is a double-edged sword. Taking microglia activation as an example, it has both positive and negative effects on ICH outcomes. In the hyperacute and acute phase of ICH, microglia activation produces a mass of pro-inflammatory factors leading to an inflammatory waterfall reaction, eventually promotes blood–brain barrier (BBB) injury, edema, and neurologic deficit. However, M2, a phenotype of microglia, improves CNS recovery by phagocytosis and secreting anti-inflammatory factors like IL-10 and CD36 (Liu et al., 2021). To sum up, the organism exerts pro-inflammatory effects in parallel with anti-inflammation in an attempt to achieve some balance after ICH.

GO/KEGG analysis indicates the functions of differentially expressed mRNAs associated with apoptosis and ferroptosis. It has been described previously that cell death improves DAMP and drives inflammatory cascades. Various types of cell death exert during ICH, including apoptosis, autophagocytosis, necrosis, necroptosis, and pyroptosis. Apoptosis is the programmed death of cells. Complex factors post-stroke lead to apoptosis, which is concerned with ATP depletion, misfolded proteins, calcium influx, free radicals, inflammatory response, α-Syn aggregation, and so on (Sekerdag et al., 2018). Red blood cells released from the ruptured vessels contain an abundance of Hb. Microglia cells phagocytize Hb, which is then metabolized into ferrous iron by ROS and lipid peroxidation. The pathological products trigger the onset of ferroptosis jointly (Wan et al., 2019).

Some typical pathways of stroke were also enriched by KEGG analysis. TNF signaling pathway has a compact relationship with microglia activation and polarization (Zhou et al., 2014). The NF-κB signaling pathway is activated within a few minutes after ICH and persists for more than a week. Accumulating evidence demonstrates that NF-κB activation leads to a large amount of pro-inflammatory cytokines release, especially TNF-α and IL-1β (Sivandzade et al., 2019). NOD-like thermal receptor protein domain associated protein 3 (NLRP3), belonging to the NOD-like receptor family, is confirmed to be a vital contributor to ICH. NLRP3 increases in microglia cells remarkably in ICH and down-regulation of NLRP3 attenuates inflammation after ICH (Yang et al., 2015). The signaling pathway of p53 contributes to apoptotic programs closely after stroke. After the stroke onset, p53 is activated immediately. Then, the activated p53 translocates to the outer membrane of mitochondria and into nuclei. On the outer membrane of mitochondria, p53 binding with B-cell lymphoma-2 (BCL-2) leads to cytosol release for the apoptotic program. In nuclei, p53 interacts with its responsive elements to initiate the transcription of apoptosis (Almeida et al., 2021). The molecules upstream or downstream of these signaling pathways may be the potential targets for the treatment of ICH.

A growing number of researchers show solicitude for the effects of non-coding RNA in pro-ICH and post-ICH. It is reported that lncRNA MEG3 participates in reducing oxidative stress and inflammation by targeting miR-181b in ICH (Xie B. et al., 2021). Apart from elevating the inflammatory level, lncRNA Blnc1 is able to mediate the permeability through the PPAR-γ/SIRT6/FoxO3 pathway in ICH (Xie L. et al., 2021). In our ce-RNA network, three significant clusters were identified. Some interactions of them have been proven to be involved in the pathological processes of a multiplicity of diseases. For instance, let-7f-5p regulated osteogenesis and bone formation by targeting TGFBR1 to ameliorate osteoporosis (Shen et al., 2019). Moreover, TGFBR1 also contributed to pulmonary fibrosis and non-small cell lung cancer (NSLC) by interacting with let-7i-5p and miR-98-5p, respectively (Jiang et al., 2019; Xu C. et al., 2022). The miR-98-5p-TGFBR1 interaction could be regulated by lncRNA Linc00511 which participated in the recurrence of NSLC (Li et al., 2022). Zhong and colleagues confirmed that miR-98-5p promoted COL1A2 inducing extracellular matrix deposition and contributing to asthma pathogenesis in human (Zhong et al., 2022). Additionally, miR-30a-5p was detected to down-regulate BCL2L11 depending on P65 for aggravating multiple myeloma (Xie et al., 2022). In Cluster 2, miR-106b-5p is a hub node. Chen and colleagues found that lncRNA H19 inhibits ICH injuries in miR-106b-5p dependent manner (Chen et al., 2021). However, the interactions among miR-106b-5p, Has2, Bcl2l11, ENSMUSG00000099757 and ENSMUSG00000075010 need further detected. While H19 is regarded as an accomplice of brain damage in another study (Mao et al., 2022). Similarly, MALAT1 inhibits inflammation and oxidative stress in ICH, as in ischemic stroke (Xu Z. et al., 2022). Through multiple post-transcriptional mechanisms, lncRNA can act as miRNA regulators or effectors, ultimately regulating the expression of mRNA in ICH. Nonetheless, the journey of finding out how lncRNA-miRNA-mRNA is involved in the physiological and pathological mechanisms of ICH is still far from over.

Compared to other studies focusing on the function of lncRNA in ICH (Cao et al., 2020; Chen et al., 2020; Xie B. et al., 2021), we employed the autologous blood injection model rather than the collagenase-induced model. The autologous blood injection model and collagenase-induced model are the most frequent and stable ICH models (Ma et al., 2011). Although neither of the two ICH models can perfectly mimic the pathophysiological situation of the disease, they have their unique advantages in simulating some processes of ICH. In the autologous blood injection model, blood enters the brain parenchyma from a single needle in a short time, which mimics the initial hemorrhage occurrence. Furthermore, the volume of the hematoma can be controlled (relating to the injected blood volume) (Kirkman et al., 2011). To investigate edema and inflammation, which is the most significant process in the acute and subacute phases, we chose the autologous blood injection model to conduct this research.

The study has several limitations that need to be addressed. Differentially expressed mRNAs and lncRNAs did not undergo functional experiments to determine their exact roles in ICH. Furthermore, the expression of differentially expressed RNAs at other phases was not investigated.

In summary, our study identified hundreds of differentially expressed mRNAs and lncRNAs in the acute ICH mouse model. Top RNA molecules of them may be the biomarker of ICH. Moreover, we analyzed the underlying relationships of lncRNA-mRNA and lncRNA-miRNA-mRNA, which may offer a potential target for ICH treatment.

## Data availability statement

The original contributions presented in the study are included in the article/Supplementary material, further inquiries can be directed to the corresponding author. The microarray data presented in the study are publicly available in the NCBI GEO database. This data can be found here: https://www.ncbi.nlm.nih.gov/geo/query/acc.cgi accession number GSE227683.

## Ethics statement

The animal study was reviewed and approved by Institutional Animal Care and Use Committee of CSU.

## Author contributions

ZY: investigation, validation, visualization, and writing—original draft. EH: investigation, validation, writing—review and editing. YC: investigation and validation. WZ: investigation. QC: visualization and software. TL: investigation. ZL: investigation. YW: supervision and writing—review and editing. TT: resources, data curation, funding acquisition, supervision, and writing—review and editing. All authors contributed to the article and approved the submitted version.

## Funding

This study was funded by grants from the National Natural Science Foundation of China (no’s. 81874425, 81973665, and 82174259); the Hunan Provincial Key Research and Development Program (2022SK2015); the Excellent Clinical Talent Training Project of SATCM (2022-543313); the Leadership Training Project of HNATCM (2022–24); the Shennong Youth Scholar Project of Hunan (2022). The content is solely the responsibility of the authors and does not necessarily represent the official views of the funding agencies.

## Conflict of interest

The authors declare that the research was conducted in the absence of any commercial or financial relationships that could be construed as a potential conflict of interest.

## Publisher’s note

All claims expressed in this article are solely those of the authors and do not necessarily represent those of their affiliated organizations, or those of the publisher, the editors and the reviewers. Any product that may be evaluated in this article, or claim that may be made by its manufacturer, is not guaranteed or endorsed by the publisher.

## Footnotes

^1^ http://www.sangerbox.com/tool

^2^ http://www.microrna.gr/LncBase

^3^ https://mirdb.org
