# Supplementary material for: mRNA and lncRNA co-expression network in mice of acute intracerebral hemorrhage
Source: Front Mol Neurosci. 2023 Apr 28;16:1166875. doi: 10.3389/fnmol.2023.1166875 (PMC10175784; doi:10.3389/fnmol.2023.1166875)
Supplement: Supplementary file 1 [file Table_1.DOCX]

Supplementary Material

mRNA and lncRNA co-expression network in mice of acute intracerebral hemorrhage

Zhe Yu^*^, Yiqing Cai, Wenxin Zhu, Quan Chen, Teng Li, En Hu, Zhilin Li, Yang Wang

*** Correspondence:** Tao Tang: tangtaotay@csu.edu.cn

# Supplementary Tables

**Supplementary Table 1.** RNA quantification and quality

| **Sample ID** | **OD260/280 Ratio** | **OD260/230 Ratio** | **Concentration (ng/μl)** | **Volume (μl)** | **Quantity (ng)** |
| --- | --- | --- | --- | --- | --- |
| 111 | 1.99 | 2.30 | 595.19 | 20 | 11903.80 |
| 112 | 1.99 | 2.37 | 873.14 | 20 | 17462.80 |
| 113 | 1.98 | 2.39 | 652.39 | 30 | 19571.70 |
| 114 | 1.99 | 2.38 | 716.01 | 30 | 21480.30 |
| 115 | 1.99 | 2.26 | 753.12 | 40 | 30124.80 |
| 122 | 1.95 | 2.32 | 599.06 | 15 | 8985.90 |
| 123 | 1.99 | 2.12 | 644.29 | 20 | 12885.80 |
| 124 | 2.01 | 2.39 | 889.00 | 30 | 26670.00 |
| 125 | 1.94 | 2.41 | 539.76 | 40 | 21590.40 |
| 126 | 2.00 | 2.39 | 869.07 | 40 | 34762.80 |

**Supplementary Table 2.** Labeling efficiency quantification and quality

| **Sample ID** | **Dye Name** | **Dye pmol/μl** | **cRNA Concentration (μg/μl)** | **Specific Activity*(pmol Dye/μg cRNA)** | **Volume**  **(μl)** | **Total Amout (μg)** |
| --- | --- | --- | --- | --- | --- | --- |
| 111 | Cy3 | 9.05 | 0.39 | 23.45 | 20.00 | 7.72 |
| 112 | Cy3 | 15.84 | 0.53 | 29.91 | 20.00 | 10.59 |
| 113 | Cy3 | 9.88 | 0.41 | 24.22 | 20.00 | 8.16 |
| 114 | Cy3 | 9.57 | 0.39 | 24.78 | 20.00 | 7.73 |
| 115 | Cy3 | 8.96 | 0.37 | 24.48 | 20.00 | 7.32 |
| 122 | Cy3 | 9.82 | 0.41 | 24.23 | 20.00 | 8.10 |
| 123 | Cy3 | 9.78 | 0.41 | 24.02 | 20.00 | 8.14 |
| 124 | Cy3 | 9.96 | 0.41 | 24.21 | 20.00 | 8.23 |
| 125 | Cy3 | 9.84 | 0.40 | 24.52 | 20.00 | 8.03 |
| 126 | Cy3 | 10.22 | 0.41 | 24.92 | 20.00 | 8.20 |

* $S\mathrm{pecific} A\mathrm{ctivity}=\frac{(pmol per \mu l d\mathrm{ye})}{(\mu g per \mu l cRNA)}$

**Supplementary Table 3.** Human homologous genes with mouse lncRNAs

| **Gene ID** | **Homologous gene**  **(Ensembl accession)** | **Length** | **Query cover** | **E value** | **Percent identity** |
| --- | --- | --- | --- | --- | --- |
| ENSMUSG00000087694 | ENSG00000250007 | 3088 | 11% | 1E-20 | 76.43% |
| AF177020 | ENSG00000286214 | 7947 | 95% | 6E-75 | 75.79% |
| ENSMUSG00000026656 | ENSG00000289273 | 864 | 1% | 6E-19 | 71.43% |
| AK041738 | ENSG00000287661 | 1861 | 1% | 5E-06 | 100.00% |
| ENSMUSG00000050708 | ITGA9-AS1-231 | 3917 | 2% | 2E-05 | 100.00% |
| ENSMUSG00000086150 | ENSG00000226455 | 876 | 51% | 0 | 84.42% |
